# Research Note: Intestinal morphology and gene expression changes in broilers supplemented with lysolecithin

**DOI:** 10.1016/j.psj.2021.101192

**Published:** 2021-04-11

**Authors:** S.E. Cloft, M. Jia, E.A. Wong

**Affiliations:** Department of Animal and Poultry Sciences, Virginia Tech, Blacksburg, VA 24061, USA

**Keywords:** soybean oil, lysolecithin, jejunum, gene expression, broiler chickens

## Abstract

Lysolecithin is used as a feed additive to aid fat digestion and absorption in broiler chickens. Previous research has shown that dietary fat source influences how broilers respond to lysolecithin supplementation. Therefore, the objective of this study was to investigate the effect of lysolecithin on a diet formulated with soybean oil on jejunum morphology and expression of selected genes in broiler chickens. Male Cobb 500 chickens were fed a Control diet or the Control diet supplemented with lysolecithin (**TRT**) from day of hatch to day 28. Jejunal samples were collected at day 10 for morphological and gene expression analysis. Feeding the TRT diet did not affect BW, villus height (**VH**), crypt depth (**CD**) or VH/CD ratio compared to Control fed chickens. Differential gene expression in the jejunum was analyzed using a custom microarray. Using a *t* test, 36 genes were found to be upregulated in TRT fed chickens compared to chickens fed the Control diet. The two most upregulated genes were carbonic anhydrase VII and interleukin 8-like 2, which are associated with healthy intestines. In summary, lysolecithin supplementation in a diet formulated with soybean oil caused no morphological changes but upregulated a number of genes in the jejunum.

## INTRODUCTION

Broiler chicks have limited ability to digest dietary fats due to reduced bile salt and lipase secretion until their gastrointestinal tract matures around 10 to 14 days of age (Wealleans et al., 2020). During this period of limitation, broilers have high energy demands, which require supplementation of dietary fat sources. Fat utilization in broiler chicks can be increased by supplementing bile salts or emulsifying agents such as lysolecithin (Zhang et al., 2011).

Lysolecithin acts to improve the health and performance of the intestine, leading to better fat and oil absorption and increased performance of broiler chickens. There was, however, no statistical link between type of dietary fat or oil and the growth response to lysolecithin addition in a large meta-analysis study conducted by Wealleans et al. (2020). Until recently the beneficial effects of lysolecithin in broilers were thought to be due to lysophosphatidylcholine (**LPC**). Brautigan et al. (2017) compared diets containing a blend of animal, poultry and vegetable fats and oils and supplemented with 2 levels of lysolecithin or the equivalent level of purified LPC. The diet containing the highest level of lysolecithin increased villus height and upregulated gene expression to a greater extent compared to the diet containing purified LPC. This result suggested that a combined effect from multiple lysophospholipids contained within lysolecithin and not LPC accounted for the improvements in intestinal performance.

The objective of this study was to investigate the effect of a diet formulated with soybean oil and supplemented with lysolecithin on jejunum morphology and expression of a selected set of genes that had been previously shown to be upregulated by lysolecithin in broiler chickens.

## MATERIALS AND METHODS

### Animals, Body Weight, and Intestinal Morphology

The experimental design was similar to that used in the study by Brautigan et al. (2017). The trial was originally designed to investigate short-term (10 d) and long-term (28 d) effects of dietary supplementation with lysolecithin. In this study, only the short-term effects were examined to provide a direct comparison with the short-term effects reported by Brautigan et al. (2017). All experimental procedures for this study were reviewed and approved by Virginia Tech's Institutional Animal Care and Use Committee. Sixty-nine day of hatch (doh) male Cobb 500 broiler chicks were obtained from the Cobb hatchery in Wadesboro, NC and transported to the Virginia Tech Poultry Research Center. Nine chicks were randomly selected for doh sampling, while the other 60 chicks were split evenly into two floor pens containing clean litter, bell waterers and feeders, separated by wire fencing. One pen was provided with a Control diet and the other pen was provided with the TRT diet that contained a lysolecithin product (Lysoforte, Kemin Industries, Des Moines, IA) supplemented at a rate of 1.0 g/kg on top of the Control diet (Table 1). This high dose of lysolecithin was used in this study because it had been shown to increase villus height and cause the greatest fold increase in gene expression in the study by Brautigan et al. (2017). The lysolecithin product was produced by enzymatically treating soybean lecithin with phospholipase to create lysolecithin. From doh until d 14, chicks were fed mash starter diets. After day 14, chickens were provided a mash grower diet prepared the same way as the starter diet and grown to day 28. On d 10, 14, 21, and 28, all chickens were individually weighed. Chickens were weighed to assess growth rates but not to generate statistically significant growth data.

**Table 1:** Ingredient and nutritional composition of the control diet.

Ingredient (%)	Starter
Corn	58.79
Soybean meal 48	29.94
DDGS	4.91
Poultry byproduct meal	2.00
Soybean oil	0.20
Salt	0.26
Sodium bicarbonate	0.15
DL Methionine	0.34
L-Threonine	0.12
L-Lysine-HCl	0.36
Limestone	1.03
Dicalcium Phosphate	1.07
Choline chloride 60	0.10
Vitamin and mineral premix	0.63
Phytase	500 ftu
Calculated Nutrient Composition (%)	
Crude protein	22.44
Poult ME kcal/kg	2960
Calcium	0.90
Avail Phosphorus	0.45
Digest Met+Cys	0.95
Digest Lys	1.28
Digest Thr	0.86

On d 10, 9 chickens per treatment were selected for sampling to ensure that there were 8 quality samples for gene expression and morphology analyses. Selected chickens were euthanized by cervical dislocation and jejunum samples were immediately collected. Jejunum samples were rinsed in 1X phosphate-buffered saline, then 3 cm segments from the center were cut and placed in neutral buffered formalin for morphology analysis. Additionally, 1.5 cm on either side of the central segment were collected and frozen for gene expression analysis. After 24 h, intestinal segments for morphology analysis were transferred to and stored in 70% ethanol prior to embedding.

Formalin fixed samples from day 10 (n = 8 per treatment) were shipped to Histo-Scientific Research Labs (Mount Jackson, VA) for embedding in paraffin. Villus height (**VH**) and crypt depth (**CD**) were measured in formalin-fixed paraffin embedded tissues. CD (n = 25/sample) and VH (n = 25/sample) were measured using Image J from the National Institutes of Health.

### Gene Expression and Statistical Analysis

Total RNA was isolated from day 10 samples (n = 8 per treatment) using TriReagent and the Directzol RNA mini prep columns (Zymo Research, Irvine, CA). cDNA was synthesized using the Qiagen RT^2^ First Strand kit. Gene expression was determined by qPCR using the Qiagen RT^2^ SYBR Green Mastermix and a custom RT^2^ profiler PCR array (CAPA 9600-12:CAPG13528D), which contained 93 genes upregulated by lysolecithin or purified LPC at d 10 in the study by Brautigan et al. (2017). The geometric mean of 3 reference genes GAPDH (glyceraldehyde-3-phosphate dehydrogenase), RPL4 (ribosomal protein L4), and RPLP1 (ribosomal protein large P1) on the array were used to calculate ΔCt. The ΔCt of the Control was used as the calibrator for calculating ΔΔCt for TRT and fold change relative to Control was calculated using the 2^−ΔΔCT^ method as described previously (Brautigan et al., 2017). Only day 10 samples were analyzed in order to directly compare expression profiles of genes in the d 10 samples of Brautigan et al. (2017) using a custom microarray, which contained only genes upregulated at d 10 and thus was only appropriate for analysis of d 10 samples.

Gene expression data were analyzed by Student's *t* test using JMP v14.0 (SAS Institute Inc., Cary, NC). Chick was considered as the experimental unit. Morphological data did not meet conditions of normal distribution and were analyzed using the nonparametric Welch's test. Statistical significance was considered when *P* ≤ 0.05.

## RESULTS AND DISCUSSION

### Body Weight and Morphological Analyses

Chickens fed the TRT diet showed no significant difference in BW compared to chickens fed the control diet throughout the trial (data not shown). There were no differences in VH, CD, or VH/CD between TRT and Control fed chickens after 10 days of supplementation (data not shown).

### Gene Expression Analysis

**Figure 1:**
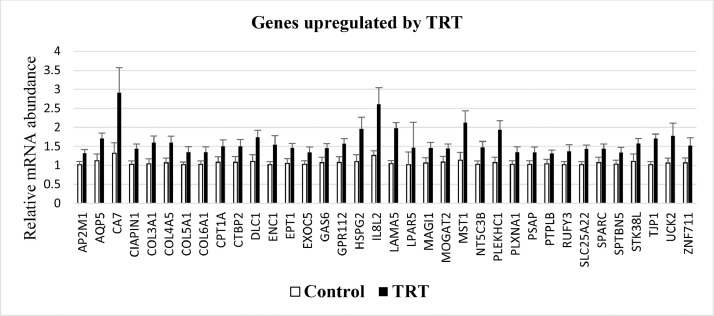
Relative mRNA expression of genes in the jejunum of chickens fed a Control diet or lysolecithin supplemented diet (TRT) for 10 d. All genes were significantly different between Control (white bars) and TRT (black bars) using a *t* test (*P* ≤ 0.05).

Chickens fed the TRT diet had 36 of 93 genes on the custom microarray significantly (*P* ≤ 0.05) upregulated compared to chickens fed the Control diet (Figure 1). There were an additional 22 genes that showed a numerical (0.06 ≤ *P* ≤ 0.10), but not statistically significant difference (data not shown).

Of the 36 genes that were statistically upregulated, 9 genes (COL5A1, CTBP2, DLC1, ENC1, GAS6, HSPG2, LAMA5, PLEKHC1, SPARC) also appeared on the list of the 28 most upregulated genes following treatment with lysolecithin or LPC (Brautigan et al., 2017). Additionally, 10 of 22 numerically upregulated genes were also on the list of the 28 most upregulated genes. The magnitude of upregulation of gene expression in the current study was much less than that in the Brautigan et al. (2017) study. In the latter study, 19 of the 28 genes showed a greater than10-fold upregulation, whereas in the current study the greatest upregulation observed was only 2.2-fold. The average upregulation of genes in the current study was only 0.5-fold.

The two most upregulated genes in TRT chickens were carbonic anhydrase VII (CA7) and interleukin 8-like 2 (IL8L2). Both genes were similarly upregulated in the current study and the previous study by Brautigan et al. (2017). CA7 was upregulated 2.2-fold in the current study and 2.6-fold in the previous study, while IL8L2 was upregulated 2.1-fold in the current study and 1.8-fold in the previous study. CA7 is associated with metabolism and was upregulated in the large intestine of 35-day-old broiler chickens following in ovo delivery of prebiotics (Slawinska et al., 2016). IL8L2 is a positive regulator of intestinal immune competence in young chickens (Bar-Shira and Friedman, 2006). Upregulation of these genes, as well as upregulation albeit to a lesser extent of many other genes by lysolecithin supplementation suggests improvement in intestinal health and competence in broilers as reported in Brautigan et al. (2017).

Brautigan et al. (2017) utilized a similar experimental design and lysolecithin inclusion rate as the current study, but their diets used a stabilized fat product containing a blend of animal, poultry, and vegetable fats and oils, whereas in the current study soy oil was the primary dietary fat source. Additionally, there was a higher total fat content in the Brautigan et al. (2017) starter diet. The different dietary fat sources lead to different fatty acid profiles between the diets; in the current study there was an estimated unsaturated to saturated fatty acid ratio (U:S) of 86%:14%, while the Brautigan et al. (2017) study had an estimated U:S of 72.9%:25.4% with 1.7% classified as other types. In contrast, the metabolizable energy of the diets was the same. Metabolizable energy values of fats are determined by their digestibility, which is dependent on the length of the carbon chain and the degree of saturation. The major difference between the current study and Brautigan et al. (2017) is that the main dietary fat source used in the diet required a larger inclusion percentage to achieve the target metabolizable energy value compared to the amount of soy oil used in the current study, likely due to the fatty acid profile differences described above.

Previous research has established that dietary fat source is an influential factor in the biological response to lysolecithin supplementation. Zhang et al. (2011) described how lysolecithin supplementation improved digestibility of linoleic and linolenic fatty acids in all dietary fat types, but only observed increased BW gain in broilers fed animal-derived fat sources rather than those fed diets containing soy oil. Lysolecithins aid fat digestion by enhancing emulsification of fats resulting in more efficient break down by lipase (Schwarzer and Adams, 1996). Unsaturated fatty acids readily form micelles, increasing their absorption compared to saturated fatty acids, especially long chain saturated fatty acids. Soybean oil is reported to be highly digestible for broilers, in part due to its high unsaturated fatty acid profile (Tancharoenrat et al., 2014). In Jansen et al. (2015), the variation in responses to lysolecithin supplementation was directly attributed to the different U:S in pig lard and soy oil. Therefore, the stabilized fat fed in Brautigan et al. (2017) with its greater amounts of saturated fatty acids and overall total fat content may have provided more opportunity for lysolecithin activity to improve intestinal activity in Brautigan et al. (2017) compared to the current study.

The effect of lysolecithin supplementation on intestinal morphology has been examined in diets formulated with different fat sources. Boontiam et al. (2017) reported that lysolecithin supplementation (0.05–0.15%) in diets containing soybean oil but with reduced metabolizable energy and essential amino acids showed no difference in both duodenal and jejunal VH. Using a diet containing the animal based fat white grease, Chen et al. (2019) showed that lysolecithin supplementation (0.025–0.075%) increased jejunal VH. Brautigan et al. (2017) reported that lysolecithin supplementation (1,000 g/T) to diets containing a combination of animal and vegetable fats increased VH and mRNA abundance of collagen genes and collagen staining in the jejunal villi. In addition to this increase in VH, lysolecithin supplementation upregulated the mRNA for seven collagen genes COL12A1, COL5A1, COL1A2, COL1A1, COL4A5, COL6A1, and COL3A1 by 17.1-, 16.6-, 12.4- 10.1-, 9.8-, 9.4-, and 7.7-fold, respectively. In the current study with soybean oil, lysolecithin supplementation (1 g/kg) increased COL3A1 and COL4A5 mRNA only 1.5-fold and COL5A1 and COL6A1 mRNA only 1.3-fold. The other collagen genes showed no differences between lysolecithin supplementation and control. This greatly reduced upregulation of mRNA for the collagen genes likely is the basis for the observed lack of increase in VH with lysolecithin supplementation in this study and may be in part due to the dietary fat source, composition and percentage inclusion used.

In summary, lysolecithin supplementation to diets containing soy oil as the fat source caused the upregulation of 36 genes in a custom microarray in the jejunum of broilers at 10 d of age. The fold increase for most of these genes was less than 2-fold. The two genes upregulated to the greatest extent in the current study were related to metabolic processes (CA7) and the development of immunological competence (IL8L2) indicating a beneficial response to lysolecithin supplementation to the diet. There was no change in VH, which is likely related to the minimal (1.3- to 1.5-fold) increase in mRNA abundance of some collagen genes. These results reflect the differences in the source and composition of dietary fat between the current and previous trials.

## DISCLOSURES

Kemin Industries, Inc provided funds and the lysolecithin product used for this research.

## References

[1] Bar-Shira, E., and A. Friedman. 2006. Development and adaptations of innate immunity in the gastrointestinal tract of the newly hatched chick. Dev. Comp. Immunol. 30:930–941. 10.1016/j.dci.2005.12.002 16430960

[bib0002] Boontiam W., Jung B., Kim Y.Y. (2017). Effects of lysophospholipid supplementation to lower nutrient diets on growth performance, intestinal morphology, and blood metabolites in broiler chickens. Poult. Sci..

[bib0003] Brautigan D.L., Li R., Kubicka E., Turner S.D., Garcia J.S., Weintraut M.L., Wong E.A. (2017). Lysolecithin as feed additive enhances collagen expression and villus length in the jejunum of broiler chickens. Poult. Sci..

[bib0004] Chen C., Jung B., Kim W.K. (2019). Effects of lysophospholipid on growth performance, carcass yield, intestinal development, and bone quality in broilers. Poult. Sci..

[bib0005] Jansen M., Nugyens F., Buyse J., Leleu S., Van Campenhout L. (2015). Interaction between fat type and lysolecithin supplementation in broiler feeds. Poult. Sci..

[bib0006] Schwarzer K., Adams C.A. (1996). The influence of specific phospholipids as absorption enhancer in animal nutrition European. J. Lipid Sci. Technol..

[bib0007] Slawinska A., Plowiec A., Siwek M., Jaroszewski M., Bednarczyk M. (2016). Long-term transcriptomic effects of prebiotics and synbiotics delivered *in ovo* in broiler chickens. PloS one.

[bib0008] Tancharoenrat P., Ravindran V., Zaefarian F., Ravindran G. (2014). Digestion of fat and fatty acids along the gastrointestinal tract of broiler chickens. Poult. Sci..

[bib0009] Wealleans A.L., Jansen M., Di Benedetto M. (2020). The addition of lysolecithin to broiler diets improves growth performance across fat levels and sources: a meta-analysis of 33 trials. Brit. Poult. Sci..

[bib0010] Zhang B., Haitao L., Zhao D., Guo Y., Barri A. (2011). Effect of fat type and lysophosphatidylcholine addition to broiler diets on performance, apparent digestibility of fatty acids, and apparent metabolizable energy content. Anim. Feed Sci. Technol..

